# Longitudinal Virological and Immunological Profile in a Case of Human Monkeypox Infection

**DOI:** 10.1093/ofid/ofac569

**Published:** 2022-11-01

**Authors:** Maria Antonella Zingaropoli, Alberico Parente, Blerta Kertusha, Roberta Campagna, Tiziana Tieghi, Silvia Garattini, Raffaella Marocco, Anna Carraro, Eeva Tortellini, Mariasilvia Guardiani, Federica Dominelli, Ombretta Turriziani, Maria Rosa Ciardi, Claudio Maria Mastroianni, Cosmo Del Borgo, Miriam Lichtner

**Affiliations:** Department of Public Health and Infectious Diseases, Sapienza University of Rome, Rome, Italy; Department of Public Health and Infectious Diseases, Sapienza University of Rome, Rome, Italy; Infectious Diseases Unit, SM Goretti Hospital, Sapienza University of Rome, Latina, Italy; Department of Molecular Medicine, Sapienza University of Rome, Rome, Italy; Infectious Diseases Unit, SM Goretti Hospital, Sapienza University of Rome, Latina, Italy; Department of Public Health and Infectious Diseases, Sapienza University of Rome, Rome, Italy; Infectious Diseases Unit, SM Goretti Hospital, Sapienza University of Rome, Latina, Italy; Department of Public Health and Infectious Diseases, Sapienza University of Rome, Rome, Italy; Department of Public Health and Infectious Diseases, Sapienza University of Rome, Rome, Italy; Department of Public Health and Infectious Diseases, Sapienza University of Rome, Rome, Italy; Department of Public Health and Infectious Diseases, Sapienza University of Rome, Rome, Italy; Department of Molecular Medicine, Sapienza University of Rome, Rome, Italy; Department of Public Health and Infectious Diseases, Sapienza University of Rome, Rome, Italy; Department of Public Health and Infectious Diseases, Sapienza University of Rome, Rome, Italy; Infectious Diseases Unit, SM Goretti Hospital, Sapienza University of Rome, Latina, Italy; Infectious Diseases Unit, SM Goretti Hospital, Sapienza University of Rome, Latina, Italy; Department of Neurosciences, Mental Health, and Sense Organs, NESMOS, University of Rome, Rome, Italy

**Keywords:** MPXV, NK cells, flow cytometry, immunophenotyping, proctitis

## Abstract

In a male with severe proctitis, monkeypox virus DNA was detected in skin lesions, blood, the nasopharynx, and the rectum, underlying generalized viral spreading. Rectal involvement was still found when skin lesions disappeared. At this early stage, an increase of cytotoxic and activated T cells was observed, while a reduction in CD56dimCD57+ NK cells compared with recovery time point was observed.

Since the eradication of smallpox, monkeypox virus (MPXV) has assumed the role of the most prominent *Orthopoxvirus* affecting human communities [1]. MPXV is often not life-threatening, although atypical and severe forms can occur in children and immunosuppressed people, suggesting an important role of the immune system in controlling the spread of disease. Here, we report a monkeypox case observed in an Italian young adult male who has sex with men reporting proctitis, in which we characterized the virological and immunological profile in different phases of the disease.

## CASE PRESENTATION

**Figure 1.**
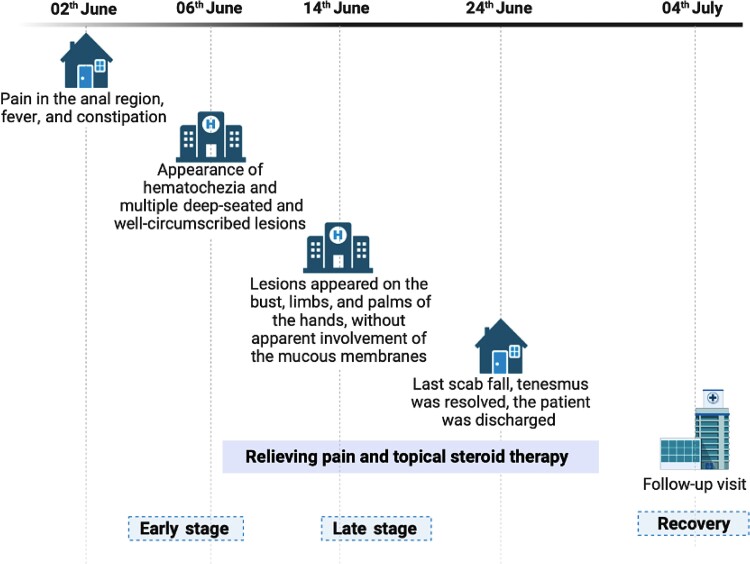
Timeline.

A 39-year-old man who has sex with men presented at the emergency department with severe anal pain, fever, constipation, and hematochezia. On physical examination, the patient was in fair general condition, pyretic, eupneic on room air, and had superficial lymph nodes that were palpable only in the inguinal area. No history of recent travel was documented. He reported last receptive anal sex 10 days before. The day after admission, 5 deep-seated and well-circumscribed lesions with central umbilication on the face and on the palms of both hands appeared. An MPXV infection was suspected, and the patient was hospitalized in the infectious diseases unit. The day after, 7 more lesions appeared on the bust, limbs, and palms of the hands, without apparent involvement of the mucous membranes. The proctitis was managed with opioid-based intravenous therapy, oral mesalazine, and a topical anti-inflammatory. The blood and microbiological tests performed are shown in Table 1; other sexually transmitted infections were ruled out. After 10 days, the last scab fell, tenesmus was resolved, and the patient was discharged and advised to abstain from sexual intercourse for 12 weeks. Subsequently, the patient underwent a colonoscopy showing anorectal congestion. Thirty days after the onset of disease, the patient returned to our clinic for a checkup, reporting well-being. The timeline is shown in Figure 1.

**Table 1.** Blood Tests and Microbiological Evaluation at the Early Stage of the Disease

	Early Stage of the Disease
WBC, cells/mmc	9640
Neutrophils, cells/mmc	5500
Lymphocytes, cells/mmc	2660
Monocytes, cells/mmc	1250
Eosinophils, cells/mmc	150
Basophils, cells/mmc	80
CRP, mg/dL	6.74
HAV IgG	Negative
HBV	
HBsAg	Negative
HBcAb	Negative
HBsAb	Negative
Anti-HCV	Negative
HIV Ab/Ag	Negative
CMV	
IgG	Positive
IgM	Negative
EBV	
EBNA IgG	Positive
VCA IgG	Positive
VCA IgM	Negative
*Treponema pallidum*	
VDRL	Negative
TPHA	Negative
Measles	
IgG	Positive
IgM	Negative
Chickenpox	
IgG	Positive
IgM	Negative
HSV-1 IgG	Positive
HSV-2 IgG	Negative
HSV-1/2 IgM	Negative
Rectal swab	
*Chlamydia trachomatis*	Not detected
*Neisseria gonorrhoeae*	Not detected

Abbreviations: Ab, antibody; Ag, antigen, CMV, cytomegalovirus; CRP, C-reactive protein; EBV, Epstein-Barr virus; HAV, hepatitis A virus; HBV, hepatitis B virus; HCV, hepatitis C virus; HSV-1, herpes simplex-1; HSV-2, herpes simplex-2; Ig, immunoglobulin; TPHA, *Treponema pallidum* hemagglutination assay; VDRL, Venereal Disease Reference Laboratory; WBC, whole blood cells.

## DIAGNOSIS

To diagnose MPXV infection, on hospital admission, samples obtained from skin lesions and the nasopharynx were sent to the National Reference Center for Infectious Disease Emergencies. Viral DNA extraction and amplification were performed as published by Antinori et al. [2]. All samples were positive for MPXV DNA on real-time polymerase chain reaction (PCR), and diagnosis of MPXV infection was made.

## VIRAL INVESTIGATION AND IMMUNOPHENOTYPING PERIPHERAL BLOOD CELL ANALYSIS

**Figure 2.**
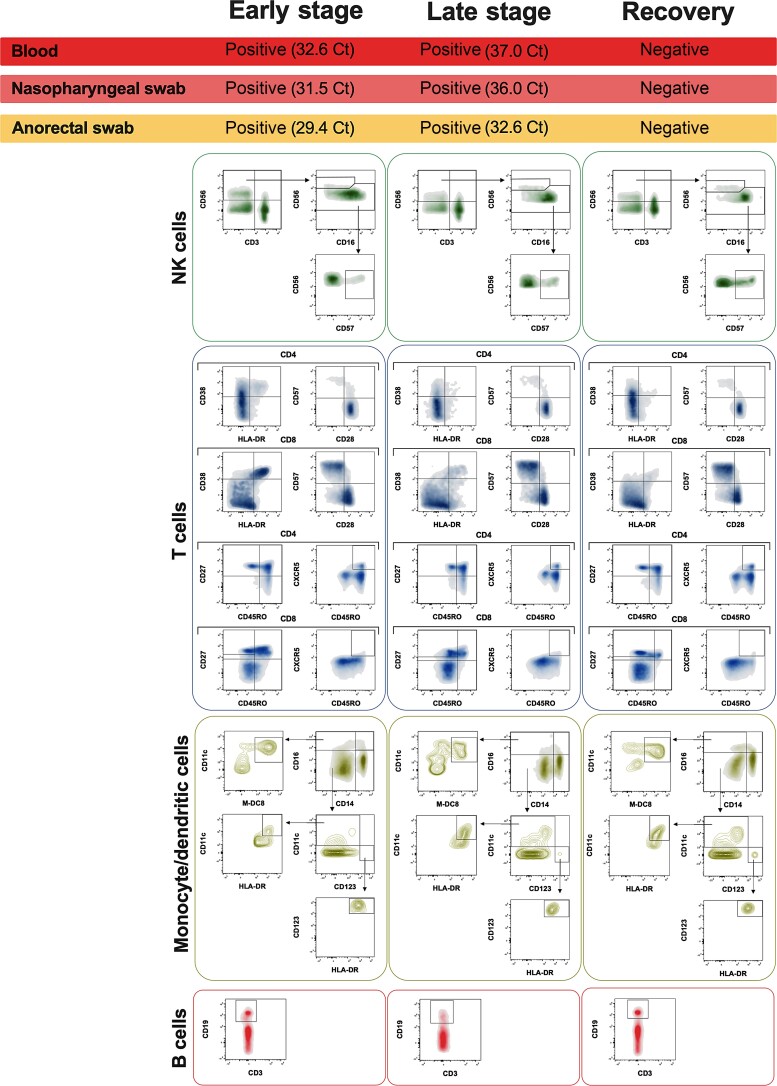
Virological and immunological evaluation over time. The longitudinal evaluation of MPXV DNA was performed by real-time PCR on blood samples, nasopharyngeal swabs, and anorectal swabs. Ct values are reported. The percentages of NK cell (CD56, CD16, and CD57), T cell (CD3, CD4 and CD8, CD27 and CD45RO), Tfh (CD45RO and CXCR5), and monocyte/macrophage markers (HLA-DR, CD14, CD16, CD11c, and CD123) were investigated. As co-expression of CD38 and HLA-DR is the key phenotype of the activation of CD4+ and CD8+ T cells in response to viral infections, we analyzed the co-expression of CD38 and HLA-DR. The immunosenescent T-cell phenotype was evaluated as a lack of CD28 expression and expression of the senescence marker CD57 [3]. Abbreviations: Ct, cycle threshold; MPXV, monkeypox virus; NK, natural killer; PCR, polymerase chain reaction; Tfh, T follicular helper.

We considered 3 time points: hospital admission (early stage), 10 days from hospitalization (late stage), and 15 days after hospital discharge (recovery) (Figure 2). For all the 3 time points, whole peripheral blood samples and nasopharyngeal and anorectal swabs were collected. For all collected samples, MPXV DNA extraction was performed using the NucliSENS easyMAG total nucleic acid extractor (bioMérieux, France), according to the manufacturer's instructions. Ten microliters of extracted DNA was used for real-time PCR (RealStar Zoonotic Orthopoxvirus PCR Kit 1.0, Altona Diagnostics).

At both the early and late-stage time points, blood samples, nasopharyngeal swabs, and anorectal swabs were positive for MPXV real-time PCR, with an increase in cycle thresholds (Cts) among the 2 time points. Otherwise, evaluation of MPXV DNA on blood samples, nasopharyngeal swabs, and anorectal swabs at the recovery time point was negative (Figure 2).

As reported in Figure 2, the peripheral blood immune cell profile was evaluated by flow cytometry. Immunofluorescence staining was assessed following a lyse-and-wash protocol, as previously described [4]. Peripheral blood immune cell phenotyping of age- and sex-matched healthy donors and HIV-infected naïve patients was performed in parallel (Supplementary Table 1).

Longitudinal evaluation of immunophenotyping analysis performed at the 3 time points showed no differences in the percentage of B cells, T cells, or CD4+ and CD8+ T cells over time. Otherwise, in the early stage of the disease, an increase of total NK cells, together with a considerable reduction in the percentage of CD56dimCD57+ NK cells, was observed compared with the recovery time point.

Among T cells, in the early stage of disease, a massive increase in the percentages of immune-activated (HLA-DR+ CD38+) CD4+ and CD8+ T cells compared with the recovery time point was observed. Otherwise, a considerable increase in the percentages of immunosenescent (CD28–CD57+) CD4+ and CD8+ T cells compared with the recovery time point was observed. Notably, in the early stage of disease, evaluation of T-cell subsets according to the expression of CD45RO and CD27 showed a slight decrease in the percentages of CD4+ and CD8+ naïve T cells and an increase in the percentage of memory and effector T cells compared with the recovery time point. Finally, among monocyte subsets, a slight decrease in the percentage of intermediate (CD14++CD16+) and nonclassical monocytes (CD14+ CD16+), as well as in the percentages of mDC, pDCs, and slanDC, was found. Conversely, a decrease in the percentage of classical monocytes was observed over time. All the results, expressed as percentages, are reported in Supplementary Table 1.

## DISCUSSION

Herein, we reported a case of MPXV infection in an Italian young adult male with proctitis. MPXV infection was found to be a self-limiting disease with a benign outcome, in accordance with other cases in the literature [5]. Conversely, proctitis is not a common manifestation [5], even if recently emerging data report proctitis as a typical presentation in this current MPXV outbreak, suggesting direct viral involvement [6]. Finally, in our case, the lymphadenomegaly was not generalized, which is characteristic of MPXV disease and not of chickenpox virus [7], but only localized in the groin area, suggesting genital entrance of the virus.

The longitudinal viral analysis showed MPXV replication not only in the skin lesions but also in the nasopharynx and the rectum, underlying generalized viral spreading. Rectal involvement was found until day 10 (late stage of the disease), when skin lesions disappeared, and the patient was considered noncontagious according to international guidelines [8]. It is important to inform patients about the possibility of still infecting others through anal sexual intercourse, which should be avoided or at least condom-protected, as previously reported [9].

The dynamic changes in the percentages of peripheral blood immune cells at the early and late stages of disease compared with the recovery time point were investigated. The most relevant finding was the massive increase of total NK-cell percentages at the early stage of disease, although a considerable decrease in CD56dimCD57+ NK cells was observed compared with the recovery time point. A total of 30%–60% of CD56dim NK cells in healthy adults express CD57, which is not expressed in immature CD56bright NK cells [10, 11]. CD56dim NK cells have a greater capacity to degranulate in the presence of targets, and CD57 defines a subset of highly mature cells [12–17]. Our data are in line with the results of Song et al., who showed that MPXV infection of rhesus macaques induces massive expansion of NK cells but suppresses NK-cell function [18]. However, in our patient, we observed an increase in the percentages of CD56dimCD57+ NK cells from the late stage of disease. In future analyses, inclusion of multiple NK-cell markers will be necessary to provide a complete description of the response.

Like NK cells, CD8+ T cells are crucial to the recognition and clearance of virus-infected cells. CD8+ T cells participate in controlling viral dissemination, and the combined action of CD8+ and CD4+ T cells prevents vaccinia virus infection from becoming lethal [19, 20]. As observed in vaccinia virus infection [19, 20], in our patient at the early stage of disease, cytotoxic CD8+ T cells were significantly activated, and trafficking into the skin to fight infection could be hypothesized. Moreover, the high percentages of immune-activated T cells found in the MPXV patient were comparable to the HIV-naïve patient without MPXV infection, highlighting how immune activation could be important in MPXV-infected subjects who live with HIV. Indeed, as it is well known that HIV infection is characterized by a state of chronic activation of the immune system [21–23], and in the current global monkeypox outbreak, a high prevalence of HIV and other sexually transmitted infections has been reported [24]. Finally, the composition of T-cell subsets in the circulating blood was also largely altered too, with an expansion phase, culminating in the generation of effector T-cells, as reported in other acute viral infections [25].

Concerning monocyte/macrophage cells, at the early stage of disease a higher percentage of total monocytes and classical monocytes compared with the recovery time point was observed. This is in line with Johnson et al. [26], who showed that in MPXV-infected cynomolgus macaques, monocytes increased the most, suggesting their development and possible recruitment from regional lymph nodes and tissues to sites of infection. This phenomenon of recruitment in monocytes to the site of infection (in this case, the skin) could restrict or slow down the spread of virus and could justify the reduction in the percentages of total and classical monocytes compared with the early stage of disease that we observed over time.

In summary, our finding underlines the role of cytotoxic cells in MPXV infection, especially by NK cells. This finding is particularly intriguing given the interest in developing strategies to augment NK-cell function during MPXV infection. Thus, although speculative, we propose that CD57 might provide a marker of these “memory” NK cells in humans that will be of interest in MPXV infection to determine the outcome of the immune response.

Despite our observations being speculative, coming from only 1 clinical case, the characterization of the peripheral blood cell profile at the early and late stages of disease compared with the recovery time point could help us better understand monkeypox infection and stimulate additional research in larger cohorts of monkeypox-infected patients to characterize the immune response and viral dynamic.

## Supplementary Material

ofac569_Supplementary_Data

## Supplementary Data

Supplementary materials are available at *Open Forum Infectious Diseases* online. Consisting of data provided by the authors to benefit the reader, the posted materials are not copyedited and are the sole responsibility of the authors, so questions or comments should be addressed to the corresponding author.
