# Supplementary material for: Longitudinal Virological and Immunological Profile in a Case of Human Monkeypox Infection
Source: Open Forum Infect Dis. 2022 Nov 1;9(12):ofac569. doi: 10.1093/ofid/ofac569 (PMC9716865; doi:10.1093/ofid/ofac569)
Supplement: ofac569_Supplementary_Data [file ofac569_supplementary_data.pdf]

**Supplementary Table 1. Immunophenotyping analysis of peripheral blood cells.** A representative healthy donor (HD) and HIV-infected naïve patient matched for sex and age are reported.

| | Stage of the disease | | | | |
|---|---|---|---|---|---|
| | **Early** | **Late** | **Recovery** | **HD** | **HIV** |
| **T cells (%)** | 50.0 | 65.6 | 53.3 | 57.3 | 39.2 |
| **CD4 (%)** | 26.2 | 27.5 | 26.0 | 44.8 | 7.6 |
| **CD4 HLA-DR+CD38+ (%)** | 7.4 | 2.4 | 1.9 | 1.2 | 6.8 |
| **CD4 CD28-CD57+ (%)** | 2.6 | 3.0 | 3.6 | 0.03 | 16.6 |
| **CD4 naïve (%)** | 25.5 | 21.0 | 28.1 | 51.1 | 38.2 |
| **CD4 central memory (%)** | 58.8 | 60.5 | 53.7 | 42.5 | 43.4 |
| **CD4 effector memory (%)** | 14.1 | 15.6 | 16.1 | 5.7 | 14.9 |
| **CD4 effector (%)** | 1.6 | 3.0 | 2.1 | 0.7 | 3.3 |
| **CD4 CD45RO+CXCR5+ (%)** | 10.0 | 11.1 | 11.2 | 7.6 | 8.43 |
| **CD8 (%)** | 49.6 | 45.8 | 47.8 | 32.1 | 76.8 |
| **CD8 HLA-DR+CD38+ (%)** | 24.3 | 3.4 | 4.8 | 1.2 | 24.9 |
| **CD8 CD28-CD57+ (%)** | 38.7 | 50.9 | 49.3 | 6.2 | 29.4 |
| **CD8 naïve (%)** | 15.6 | 21.5 | 22.0 | 74.0 | 14.1 |
| **CD8 central memory (%)** | 36.8 | 15.9 | 7.6 | 15.5 | 39.7 |
| **CD8 effector memory (%)** | 13.2 | 2.4 | 2.0 | 5.3 | 25.1 |
| **CD8 effector (%)** | 30.7 | 53.1 | 62.0 | 2.2 | 13.2 |
| **CD8 intermediate (%)** | 3.7 | 7.0 | 6.3 | 2.9 | 8.3 |
| **CD8 CD45RO+CXCR5+ (%)** | 1.4 | 1.4 | 0.7 | 0.3 | 2.8 |
| **NK cells (%)** | 13.6 | 10.4 | 6.9 | 3.9 | 6.3 |
| **CD56dim (%)** | 96.0 | 95.1 | 95.8 | 94.2 | 82.8 |
| **CD56bright (%)** | 4.3 | 5.3 | 4.3 | 5.7 | 3.1 |
| **CD56dim CD57+ (%)** | 6.8 | 13.7 | 29.4 | 20.4 | 20.1 |
| **NKT cells (%)** | 1.9 | 2.9 | 6.7 | 3.2 | 2.1 |
| **B cells (%)** | 7.2 | 6.5 | 6.4 | 8.4 | 3.5 |
| **Monocytes (%)** | 24.9 | 19.1 | 16.5 | 16.5 | 31.5 |
| **Classical monocytes (%)** | 50.2 | 40.2 | 29.6 | 32.8 | 30.5 |
| **Intermediate monocytes (%)** | 1.7 | 2.1 | 3.7 | 1.2 | 3.0 |
| **Atypical monocytes (%)** | 2.7 | 2.8 | 4.3 | 4.6 | 1.8 |
| **DCs (%)** | 16.4 | 10.5 | 6.9 | 5.1 | 10.0 |
| **SlanDCs (%)** | 0.4 | 0.3 | 0.5 | 0.5 | 0.07 |
| **mDCs (%)** | 0.3 | 0.7 | 0.7 | 0.3 | 0.3 |
| **pDCs (%)** | 0.06 | 0.07 | 0.09 | 0.1 | 0.05 |

NK: natural killer, DC: dendritic cell, mDC: myeloid cell, pDC: plasmacytoid cell, HD: healthy donor, HIV: human immunodeficiency virus.
